# Supplementary material for: Machine learning models of healthcare expenditures predicting mortality: A cohort study of spousal bereaved Danish individuals
Source: PLoS One. 2023 Aug 7;18(8):e0289632. doi: 10.1371/journal.pone.0289632 (PMC10406307; doi:10.1371/journal.pone.0289632)
Supplement: S1 Fig — Points falling in the diagonal line represent perfect calibration of the model. (DOCX) [file pone.0289632.s001.docx]

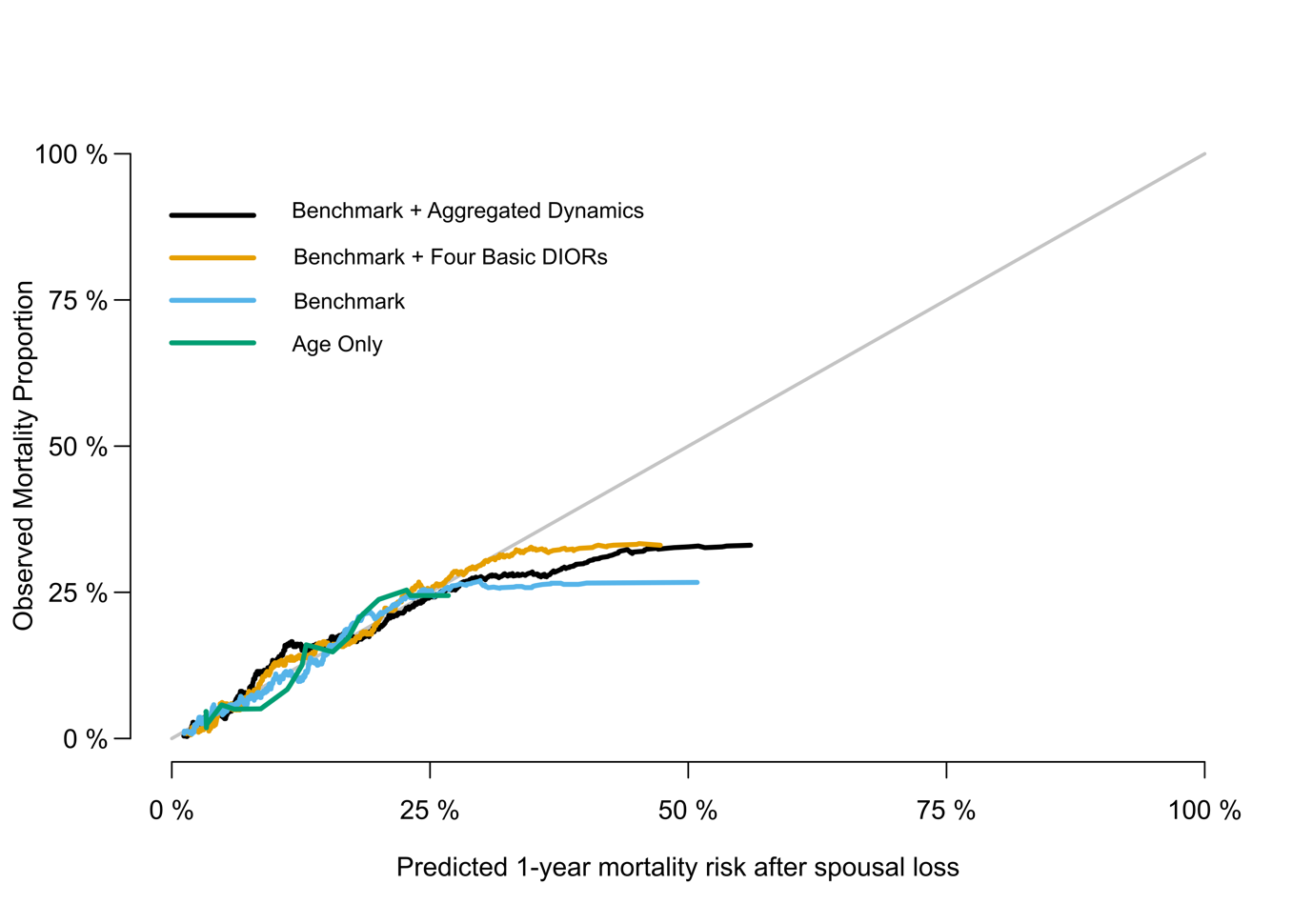


**S1 Fig. Calibration plot for males showing risk estimates of all-cause mortality within the year after spousal bereavement against outcome proportions observed in the holdout dataset.** Points falling in the diagonal line represent perfect calibration of the model
