# Supplementary material for: Machine learning models of healthcare expenditures predicting mortality: A cohort study of spousal bereaved Danish individuals
Source: PLoS One. 2023 Aug 7;18(8):e0289632. doi: 10.1371/journal.pone.0289632 (PMC10406307; doi:10.1371/journal.pone.0289632)
Supplement: S3 Fig — The x-axis for both panels A & B (‘Risk Threshold’) shows the range of risk threshold probabilities, that is the probabilities which when exceeded, individuals are classified as high risk of dying within 1-year. For panel A: The y-axis shows the “False Negative Rate” which is the proportion of false negatives among all positives. For panel B: The y-axis displays the “False Negative Rate Difference”, that is the False Negative Rate (FNR) of “All DIORs + Sociodemographics” model minus the FNR of “Sociodemographics Only” model. The shaded purple areas around the lines of both plots represent 95% confidence intervals of the FNR and the FNR difference via bootstrapping (500 samples). (DOCX) [file pone.0289632.s003.docx]

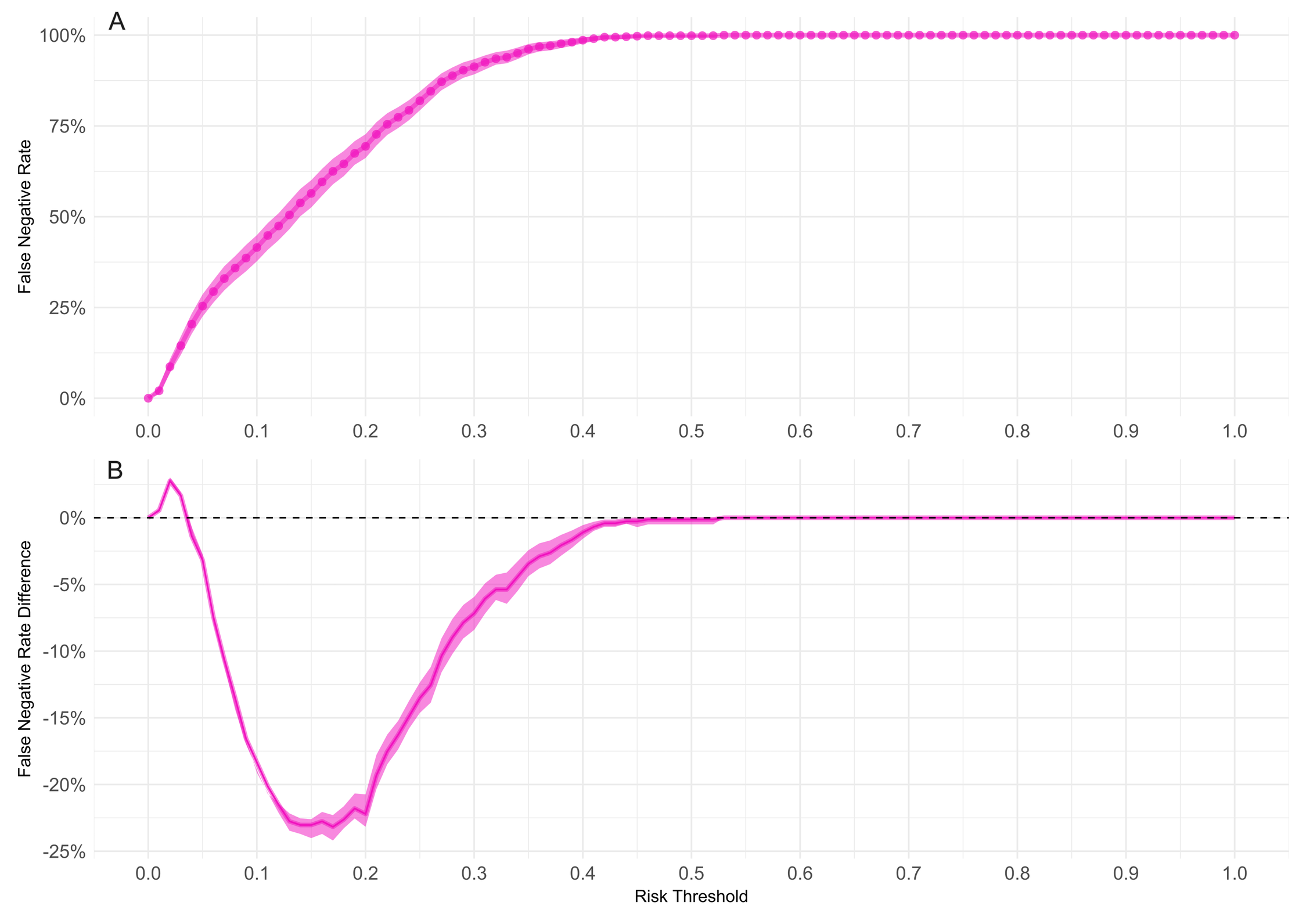


**S3 Fig. False negative rates of “All DIORs + Sociodemographics” model developed to estimate the risk of all-cause mortality within the year after spousal bereavement.** The x-axis for both panels A & B (‘Risk Threshold’) shows the range of risk threshold probabilities, that is the probabilities which when exceeded, individuals are classified as high risk of dying within 1-year. For panel A: The y-axis shows the “False Negative Rate” which is the proportion of false negatives among all positives. For panel B: The y-axis displays the “False Negative Rate Difference”, that is the False Negative Rate (FNR) of “All DIORs + Sociodemographics” model minus the FNR of “Sociodemographics Only” model. The shaded purple areas around the lines of both plots represent 95% confidence intervals of the FNR and the FNR difference via bootstrapping (500 samples).
