# Supplementary material for: Machine learning models of healthcare expenditures predicting mortality: A cohort study of spousal bereaved Danish individuals
Source: PLoS One. 2023 Aug 7;18(8):e0289632. doi: 10.1371/journal.pone.0289632 (PMC10406307; doi:10.1371/journal.pone.0289632)
Supplement: S1 Table — (DOCX) [file pone.0289632.s004.docx]

**S1 Table. Performance differences of prediction models stratified on sex in the holdout set against benchmark model**

|  | **Males** | |  | **Females** | |
| --- | --- | --- | --- | --- | --- |
|  | Delta AUC (%) | Delta Brier (%) |  | Delta AUC (%) | Delta Brier (%) |
| Age Only | -3.30 [-4.99, -1.61] | 0.08 [0.01, 0.14] |  | -4.40 [-6.20, -2.62] | 0.03 [0.00, 0.06] |
| Benchmark + 4 Basic Dynamical Indicators | 6.79 [4.45, 9.14] | -0.29 [-0.43, -0.15] |  | 9.39 [6.91, 11.87] | -0.19 [-0.26, -0.12] |
| Benchmark + Overall Trend Dynamics | 6.05 [3.58, 8.52] | -0.25 [-0.40, -0.10] |  | 9.77 [7.30, 12.25] | -0.20 [-0.28, -0.13] |
| Benchmark + Dispersion Dynamics | 5.44 [3.03, 7.84] | -0.19 [-0.35, -0.04] |  | 9.30 [6.87, 11.74] | -0.19 [-0.26, -0.12] |
| Benchmark + Memory Dynamics | 3.08 [1.17, 4.50] | -0.09 [-0.19, 0.02] |  | 6.12 [4.01, 8.23] | -0.08 [-0.13, -0.03] |
| Benchmark + Aggregated Dynamics | 6.02 [3.61, 8.44] | -0.25 [-0.41, -0.10] |  | 9.72 [7.19, 12.25] | -0.19 [-0.27, -0.10] |
| AUC: Area Under Curve  Delta AUC = AUC of each Model – AUC of Benchmark  Delta Brier = Brier Score of each Model – Brier Score of Benchmark  Brackets show the 95% confidence intervals for the difference in performance measures  The Benchmark model is used as a reference for the contrasts | | | | | |
