# Supplementary material for: Machine learning models of healthcare expenditures predicting mortality: A cohort study of spousal bereaved Danish individuals
Source: PLoS One. 2023 Aug 7;18(8):e0289632. doi: 10.1371/journal.pone.0289632 (PMC10406307; doi:10.1371/journal.pone.0289632)
Supplement: Attachment — Submitted filename: Response-to-Reviewers.docx [file pone.0289632.s006.docx]

**Response to Editor & Reviewers**

| No. | **Comments by Editor** | **Response to Editor** | **Change in the revised draft (Manuscript Draft)** |
| --- | --- | --- | --- |
| n/a | Thank you for submitting your manuscript to PLOS ONE. After careful consideration, we feel that it has merit but does not fully meet PLOS ONE’s publication criteria as it currently stands. Therefore, we invite you to submit a revised version of the manuscript that addresses the points raised during the review process. | We are very pleased to receive comments from the editor and the reviewers. We have responded to each of the points raised, as shown below. We believe that the revised draft has been substantially improved and fully addresses all concerns. | n/a |
| **1**. | The following comments might help the authors with some clarity on the manuscript: 1. What method of adding predictors to the model was used? 2. How was the affluence index created? 3. Cite the Brier score and explain it as well 4. Why was it significant/important to stratify the analysis by gender 5. P-values should be included in the tables 2 and 3 or any other measure of statistical significance to observe the statistical difference between the groups. | We thank the editor for their comments. We believe that the comments improved our work.   1. We did not use a specific method of adding predictors to the model. However, based on your comment, we now describe our statistical procedure explicitly in the Statistical Methods subsection. 2. We now provide a more detailed description of the affluence index and cite the relevant papers related to the methodology and application. 3. The Brier score is now explained and cited in the Statistical Methods section. 4. We now cite recent previous work indicating sex differences in mortality after spousal bereavement in a similar Danish cohort. In accordance to that, we proceed by developing also separate models for the two sexes, to explore potential differences in performance of the models. 5. P-values are now shown for both tables 2 and 3. We note that for Table 3, it is not possible to provide a measure of statistical difference between the groups, as the model is fitted into a different dataset each time, corresponding to the specific restricted age range. Hence statistical differences and p-values are shown for within group comparisons, where we compare in each age group, the performance of a null model (no predictors) with our DIORs model. With regards to Table 2, we constructed a supplementary table (S1 Table) displaying the differences in performance along with their respective 95% confidence intervals. | 1. Line 172 – 178 2. Line 138 – 143 3. Line 196 – 207 4. Line 106 – 108 5. (Table 2 & 3, S1 Table) |
|  | **Comments by Reviewer #1** | **Response to Reviewer #1** | **Change in the revised draft (Manuscript Draft)** |
| **1.** | 1. Conclusion should be clearly and concisely answered the main research question 2. Briefly and precisely refer to the summary of the important results 3. What was the of participation rate in the present study? 4. Table 1: provide a p-value column for comparison of variables among gender subgroup 5. Please explain more in detail about Affluence Index Group in methods section also insert a source as citation 6. Table 1: Distribution of socio-demographic variables of the study sample should be report by Test and Trained samples 7. Table 2: the column of p-value should be added. | We thank the reviewer for their comments.   1. We think that the conclusion stated in the last paragraph of the main text is both clear and concise. We made some modifications so that the conclusions of the study are better aligned with the main aim. 2. We think that the manuscript already provides a listed summary of the important results in the first paragraph of the Discussion Section. 3. The current study is a registry based nationwide cohort one, on Danish older adults. Danish citizens residing in Denmark are by default followed up in the registers via Statistics Denmark (https://www.dst.dk/da/) without any given consent. Information on the inclusion and exclusion criteria are provided in the main text of the manuscript. 4. The p-value is now added to Table 1 5. We have explained Affluence Index more in detail, also citing sources of its methodology and applications. 6. We have modified Table 1, according to your comment. 7. Table 2 has p-value indication now. In addition, we constructed a supplementary table (S1 Table) quantifying the differences in performance along with their respective 95% confidence intervals. | 1. Line 452 - 459 2. Line 364 – 376 3. Line 145 – 152 4. Table 1 5. Line 137 – 143 6. Table 1 7. Table 2 – S1 Table |
|  | **Comments by Reviewer #2** | **Response to Reviewer #2** | **Change in the revised draft (Manuscript Draft)** |
|  | The authors have presented a well-written and concise study of predicting mortality using machine learning models with features such as age, sex, sociodemographic factors, and healthcare expenditures. The authors conclude that adding healthcare expenditures as features to age and sex improves mortality predictability more than adding sociodemographic factors. | We thank the reviewer for finding the current study well-written and concise. | NA |
| **1.** | During my review of the paper, I highlighted the following areas for improvement:  The authors have presented confidence intervals for AUC and Brier scores. From my understanding of the methodology, the authors have only 1 holdout set with 25% of the data. In this case, how are the confidence intervals computed since there would be only one AUC value and Brier score per model for the holdout set? | We now include in the main text of the manuscript the process of confidence-intervals creation, as well as the computational package used to extract them. | Line 190 – 192  Line 205 - 207 |
| **2.** | It would be helpful to add whether the holdout set was created using a stratified train-test split given the label imbalance (~6% mortality rate). | The information is now included in the main text (Results Section). | Line 247 - 250 |
| **3.** | AUC is a good metric to measure model performance when the labels are fairly balanced. However, in this case, the large proportion of negative labels can result in very low false positive rates, thus affecting the AUC calculations. The author’s conclusions would be bolstered by including the comparisons of average precision (which is the area under the precision-recall curve) across the different models due to the imbalanced labels. | We agree that due to the rarity of the outcome, the average precision might be an insightful metric to present. Since the average precision is a weighted average of the proportion of positive identifications that were actually correct, it considers the number of false positives in the denominator. We believe that we have already addressed this in the current manuscript by implementing the decision curve analysis which shows the net benefit of the models, the latter computed by using both the true positives (benefits) and the false positives (harms) for a range of risk thresholds, thus being affected by the rarity of the outcome. We made changes to the manuscript to better clarify the decision curve analysis and the net benefit extracted from the aforementioned. Therefore, the predictive performance of our models has been compared and evaluated on the basis of not only the AUC, but also the Brier score (proper scoring rule) and the Net Benefit. | Line 211 – 216  Figure 5 : Made legend more explicit |
| **4.** | The authors have presented true positive rate in Fig 5 but did not provide the false negative rates of the models. Since the goal is to predict mortality, it would be suitable to understand the models’ performance on false negative rates. A model with high false negative rate would incorrectly predict no mortality for individuals with observed mortality and hence may be less suitable as a decision support tool. | As mentioned in the text and in the response above, Fig 5 does not represent the True Positive Rate, but the Net Benefit, the latter considering both the number of True Positives and False Positives. We slightly modified parts of the text to make this statement clearer to the reader (see response above). However, we do agree that it would be useful to present the false negative rate (FNR) of the models. Hence that information is now provided as part of the main text but also as supplementary figure (S3 Fig). Nonetheless, performance measures of a model have no meaning except when compared with a reference or another model. Therefore, we also examined the difference in FNR between the DIORs model and the one using only sociodemographic information, to provide evidence of DIORs increasing the accuracy of risk assessment. Hence, while we acknowledge that a model with DIORs misses mortality cases among the bereaved ones (as indicated by FNR), it misses fewer ones than the benchmark model, for a reasonable range of risk thresholds. Also compared with sociodemographic information models, the DIORs model is the one with the highest Net Benefit, thus making it more reliable under a decision support framework. | Line 211-216  Figure 5: Made legend more explicit  Line 326 – 329  Line 419 - 422  S3 Fig |
| **5.** | Line 306 - It was not clear how raw weekly expenditures were used as features. For example, were the feature values equal to expenditure in each week starting on Jan 1, 2011? In this case, what were the feature values after an individual’s death? | We agree with the reviewer that a reader might be confused with our explanations of how expenditures are used as features. We have now added this information to the main text of the draft. Additionally, in the Study Population section, we now mention the start of our follow-up, which is the date of spousal bereavement. Since it is incorrect to use information after time zero (date of bereavement) to predict the future outcome, information on healthcare expenditures for each individual was used for a period of two years (104 weeks), starting from two years before the bereavement date, and ending the week before the stressor’s event. Therefore, the starting date of monitoring expenditures was different for each individual and was dependent on their unique bereavement date. We did not include or modelled information of healthcare expenditures after the date of bereavement, and hence date of death, as that was not the aim of our analysis. | Line 150 – 152  Line 343 – 346 |
| **6.** | Fig 4 - The definitions of observed mortality proportion and predicted 1-year mortality risk could not be ascertained. Please describe the axes, similar to Fig 5. | This has now been added to Figure 4. | Fig 4. |
| **7.** | Line 122 - duplicated “date of” | Fixed. | Line 124 |
|  | **Comments by Reviewer #3** | **Response to Reviewer #3** | **Change in the revised draft (Manuscript Draft)** |
|  | The manuscript appears to be technically sound and the data presented support the conclusions drawn by the authors. The study is well-designed and the analysis methods used are appropriate. The authors provide a detailed description of the data and their methods, as well as the results obtained, which are presented in a clear and concise manner. The results are discussed in the context of the existing literature, and the limitations of the study are acknowledged. Overall, the manuscript appears to be a well-executed study that provides important insights into the prediction of all-cause mortality following spousal bereavement.   The research text describes a study aimed at comparing prognostic models of all-cause mortality risk based on healthcare expenditures data. The study uses Danish population registers data and analyzes healthcare expenditures of Danish citizens aged 65 years and above, who had been residents of Denmark for at least 5 years before their date of death, from January 1st, 2011, to December 31st, 2016. The study population is restricted to those who suffered spousal bereavement during the time window 2013-2016. The authors computed various Dynamic Indicator of Overall Risk (DIORs) based on different properties of the underlying dynamic process of healthcare expenditures. The DIORs were used as predictors in XGBoost tree-based models to predict all-cause mortality risk.  The research provides sufficient information on the study population, data sources, and analytical methods. The use of national population registers to obtain a large study population with a unique personal identifier for each citizen is a strength of the study.  The section describing healthcare expenditures and knowledge discovery provides a clear explanation of the computation of the DIORs.  The section describing the statistical methods is well-written and provides clear information on the XGBoost tree-based algorithm, the selection of hyperparameters, and the assessment of the models. The use of a 5-fold cross-validated dataset of the initial training set and a holdout set to validate the models' generalization ability is a strength of the study.  Overall, the study provides a clear description of the methods used to analyze the healthcare expenditures data and predict all-cause mortality risk. However, the lack of presentation of mathematical formulas for DIORs is a potential gap. | We are very pleased to see that the reviewer found our work as technically sound and well conducted.  We acknowledge that mathematical formulas of the DIORs are not presented in the current study. However, we cite the sources where these easily extracted metrics can be found along with their formulas, while also having our code of analysis publicly available, the latter showing the process and the packages used to extract the aforementioned. Our aim is to focus more on the applied perspective of DIORs and thus investigate their predictive capacity in the Danish registers. | NA |
